# Aseptic Venous Abscess Caused by a Foreign Body Granuloma Following Cyanoacrylate Glue Embolization for Chronic Great Saphenous Vein Reflux: A Case Report

**DOI:** 10.3400/avd.cr.26-00004

**Published:** 2026-05-16

**Authors:** Takasumi Goto, Hironobu Fujimura, Shigeru Miyagawa

**Affiliations:** 1 Department of Cardiovascular Surgery, Toyonaka Municipal Hospital, Toyonaka, Osaka, Japan; 2 Department of Cardiovascular Surgery, Osaka University Graduate School of Medicine, Suita, Osaka, Japan

**Keywords:** cyanoacrylate glue embolization, aseptic venous abscess, foreign body granuloma

## Abstract

This report describes the case of an 88-year-old male who underwent cyanoacrylate glue embolization (CAE) for right great saphenous vein (GSV) reflux. Three months postoperatively, the patient presented with phlebitis and a subcutaneous abscess along the occluded right GSV. Surgical removal of the GSV and associated skin fistula was successful. Cultures of the excised tissue showed no evidence of bacterial infection. Histological analysis demonstrated a foreign body granuloma and phlebitis complicated by an aseptic abscess. The postoperative course was uneventful, with no recurrence at 1.5 years postoperatively. Careful surveillance remains necessary to detect late complications following CAE.

## Introduction

Cyanoacrylate glue embolization (CAE) has recently gained recognition as a less invasive treatment option for venous reflux. Its efficacy in treating symptomatic venous reflux has been reported to be comparable to that of thermal ablations.^1–5)^ With a favorable safety profile and minimal complications, CAE has been established as a promising endovenous therapy for symptomatic great saphenous vein (GSV) reflux, comparable to thermal ablations.^3–5)^

The occurrence of adverse complications after CAE is relatively rare. The most common adverse event reported in previous trials was phlebitis of the occluded veins, with a prevalence rate of 5%–25%.^5,6)^ Phlebitis is mostly mild and conservatively self-limiting.^7)^ Other adverse events associated with cyanoacrylate glue include type I or IV hypersensitivity reactions, endovenous glue-induced thrombosis (EGIT), infection, and foreign body granuloma. Recently, a few cases of phlebitis following CAE, for which open surgical removal of the embolized GSV was required, have been reported.^8)^

Herein, we report a case of aseptic venous abscess following phlebitis, possibly caused by advancement of a foreign body granuloma after CAE for symptomatic GSV reflux, for which open surgical removal was subsequently required.

## Case Report

The patient, an 88-year-old male with asymptomatic chronic leukocytosis, was referred to our institution for right leg edema, fatigue, and frequent cramps. His height and weight were 152 cm and 47.8 kg, respectively. Moreover, he had a history of radiofrequency ablation (RFA) for left GSV reflux, conducted 1 year prior. Lower limb venous ultrasound findings revealed extensive venous reflux of the right GSV from the saphenous–femoral junction (SFJ) to the distal end; the diameters of the femoral GSV and crural GSV were 5.9 and 3.0 mm, respectively. Incompetent Boyd and Dodd perforating veins were also observed. Laboratory data revealed that the white blood cell count (WBC) was elevated to 20100/μL due to chronic leukocytosis; however, the C-reactive protein level (CRP) was 0.57 mg/dL. Based on these findings, the patient was diagnosed with symptomatic right GSV reflux with a Clinical–Etiology–Anatomy–Pathophysiology classification of grade C3. The patient had experienced a previous complication with a subcutaneous hematoma due to compression therapy after RFA for left GSV varix and had no history of allergies. Thus, we decided to perform CAE.

CAE (VenaSeal Closure System; Medtronic, Minneapolis, MN, USA) via the GSV around the medial malleolus was performed for right GSV reflux. There was no extravasation of the wire, and the total operative time was 23 min. On postoperative ultrasound, EGIT was class 0–I, with occlusion of all perforating branches.

**Fig. 1:**
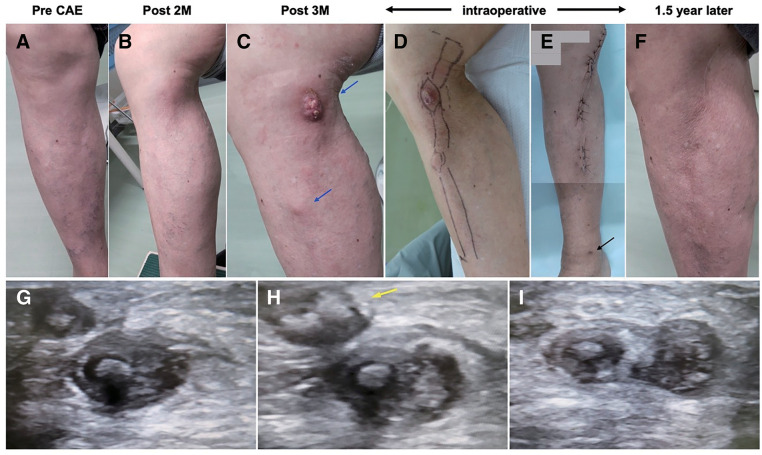
Preoperative clinical and ultrasound findings of the right lower leg. Preoperative clinical courses are shown in (**A**–**F**), and the ultrasound findings are shown in (**G**–**I**). (**A**) Before CAE. (**B**, **C**) Slight phlebitis in the right lower leg along the glue-occluded GSV 2 months after CAE. The phlebitis lesion that advanced to the venous abscess (**C**; blue arrows) and the lesion in the knee joint created a skin fistula (**C**) at 3 months postoperatively. (**D**, **E**) Preoperative and postoperative macroscopic findings. (**E**) The puncture site at the time of CAE at the right GSV around the medial malleolus (black arrow). (**F**) Infective phlebitis recurrence was not observed at 1.5 years after removal of the GSV. (**G**) The GSV is filled with a thrombus containing cyanoacrylate glue, and the vessel wall is edematous. Venous abscess is observed at the occluded GSV (**H**, **I**) along with fistula formation and a subcutaneous abscess (yellow arrow) at the puncture site. CAE: cyanoacrylate glue embolization; GSV: great saphenous vein

Two months postoperatively, mild phlebitis developed in the right lower leg along the glue-occluded GSV; however, no other symptoms were observed (**Fig. 1A** and **1B**). Postoperative phlebitis was suspected. Therefore, conservative therapy with antihistamines and topical corticosteroids was initiated. Three months postoperatively, an abscess formed gradually on the right GSV on the inside of the knee joint (**Fig. 1C**). Cultures of the white purulent discharge were all negative. Laboratory testing revealed no major abnormalities in inflammatory markers, with WBC and CRP levels of 14100/μL and 0.36 mg/dL, respectively. Furthermore, there was no significant increase in the eosinophil count (141/μL). Lower limb venous ultrasound revealed thrombophlebitis in the right lower leg GSV, while the venous abscess was connected to the skin fistula (**Fig. 1G**–**1I**). The patient was diagnosed with postoperative venous abscess following phlebitis and was subsequently readmitted.

**Fig. 2:**
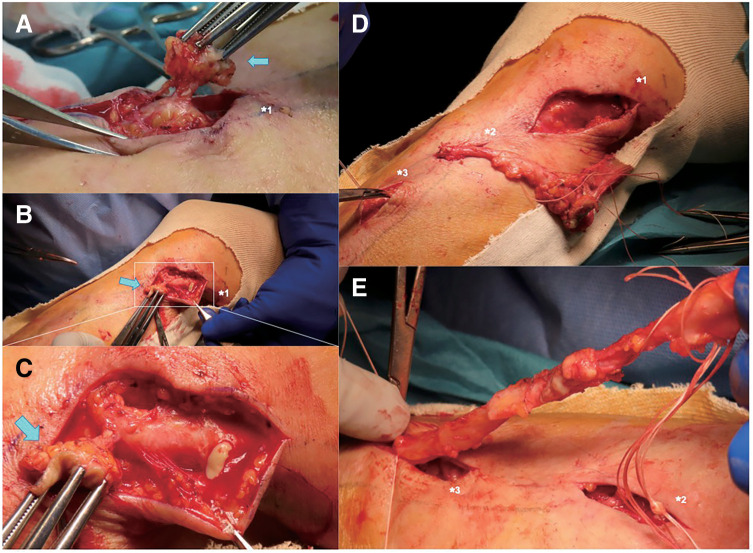
Intraoperative findings. (**A**–**C**) Complete removal of the infected GSV, including the venous abscess and skin fistula (blue arrow). (**D**, **E**) The infected GSV was pulled out through the islanded distal incisions (indicated as 1, 2, and 3) and resected. GSV: great saphenous vein

After general anesthesia, the proximal incision was placed along the right lower leg GSV, avoiding the region around the abscess (**Figs. 1D** and **2A–2C**). Strong adhesion was observed between the occluded GSV and the neighboring tissues, with the vessel lumen filled with a white, pus-like discharge (**Fig. 2C**). The exposed GSV was ligated and resected at the proximal end through the incision and subsequently pulled out from the islanded distal incisions (**Fig. 2D**). Eventually, the GSV, including the skin fistula, was removed from the distal incision, preserving the GSV in the right femoral lesion and the distal one-third of the lower leg (**Figs. 1E** and **2E**). The total operative time was 64 min.

**Fig. 3:**
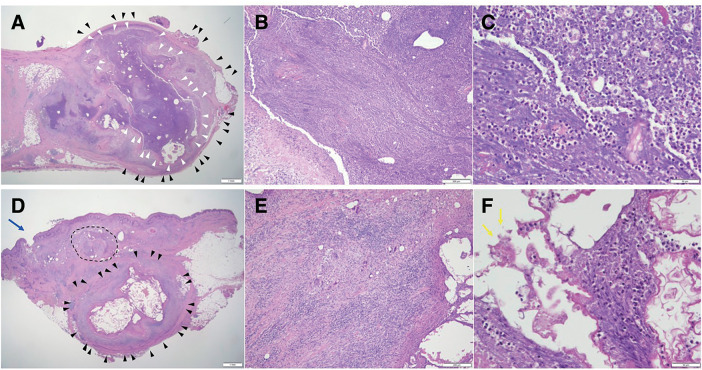
Pathohistological findings of the venous abscess and foreign body granuloma. (**A**–**C**) Hematoxylin and eosin staining of the phlebitis complicated by the abscess (**A**, **B**, and **C**: ×12.5, ×100, and ×400 with scale bars of 1 mm, 200 μm, and 50 μm, respectively). The vein was filled with abscess contents (white arrowheads), including numerous neutrophils, and the vascular endothelial cells were completely destroyed. The outline of the venous vascular wall is shown in black arrowheads. Phlebitis with aseptic venous abscess was diagnosed based on these histological findings. (**D**–**F**) Hematoxylin and eosin staining of the abscess with the skin fistula (**D**, **E**, and **F**: ×12.5, ×100, and ×400 with scale bars of 1 mm, 200 μm, and 50 μm, respectively). (**E**) The skin fistula is shown as a blue arrow, and the abscess formation, shown inside the black dotted line, mainly comprises neutrophils. A foreign body granuloma, which was a representative histological change after cyanoacrylate glue embolization, was observed (black arrowheads). Some foreign contents were observed in the vein (yellow arrows).

Postoperative antibiotic therapy (tazobactam/piperacillin 2.25 g × 3/day) was continued until postoperative day 3, after which it was discontinued as cultures of the excised vessels and discharge showed no evidence of bacterial infective phlebitis. Pathohistological analysis revealed phlebitis complicated by venous abscess and a foreign body granuloma without any bacterial findings (**Fig. 3**). Peripheral neuralgia along the scar was observed, and the patient was discharged on postoperative day 10. At 1.5 years postoperatively, recovery has been uneventful, with no recurrences of the abscess (**Fig. 1E**).

## Discussion

This case involved a subcutaneous abscess with fistula formation arising from the phlebitis of the occluded GSV, which could not be controlled conservatively. Therefore, surgical resection of the GSV, including the abscess, was required. Interestingly, so far, there has been no recurrence in the preserved GSV without any antiallergic medications, such as steroids or antihistamines. Regarding the etiology, type I and IV hypersensitivity were deemed inapplicable, given that the eosinophil count before and after CAE showed no significant increase, and phlebitis occurred 2–3 months after CAE. There was also no evidence associated with ascending thrombophlebitis since EGIT was class 0–I. There were no bacterial findings in any of the cultures of the resected vessels and discharge. Histopathological analysis revealed a foreign body reaction, which was recognized as one of the representative histological changes after CAE^6,8)^; moreover, phlebitis complicated by an abscess was observed. Accumulation of inflammatory cells, predominantly neutrophils and smaller populations of lymphocytes and macrophages, was observed (**Fig. 3**). Based on those clinical and pathohistological findings, the patient was ultimately diagnosed with noninfective phlebitis complicated by abscess formation associated with a foreign body granuloma.

Regarding potential risk factors for this aseptic venous abscess, we suspected that the patient's pre-existing chronic leukocytosis contributed to an exaggerated foreign body reaction to the cyanoacrylate glue, leading to abscess formation. The patient had a history of asymptomatic chronic leukocytosis, which was potentially reflected as a persistent low-grade inflammatory state or an altered immune response. Such an immunologic background could theoretically amplify the body’s reaction to foreign materials. Thus, we hypothesized that this heightened inflammatory response could have promoted glue encapsulation and subsequent aseptic venous abscess development. Moreover, no evidence of bacterial infection was observed in the setting of chronic inflammation, further supporting a noninfectious, immune-mediated etiology. While chronic leukocytosis is not an absolute contraindication to CAE, greater caution is warranted in patients with underlying immunologic abnormalities.

A foreign body reaction is generally observed in most patients after CAE.^6)^ Foreign body granuloma generally develops in advanced cases following a foreign body reaction or occurs as a result of type IV hypersensitivity in some patients^9)^; however, the precise mechanisms underlying the development of foreign body granuloma remain uncertain yet. Some reported cases have included asymptomatic subcutaneous masses, skin breakdown, or glue cast extrusion^6)^; however, cases of granuloma-induced abscess formation requiring surgical resection, as in our case, are extremely rare. Regarding the potential risk factors for phlebitis after CAE, supra-fascial saphenous veins located close to the skin have been identified, with a reported subcutaneous distance between the anterior vein wall and skin of <1 cm and a GSV diameter of 8 mm.^7)^ In our case, for which phlebitis was considered a risk factor, the patient experienced mild phlebitis at the right lower leg inside along the glue-occluded GSV, which was found 2 months after CAE. The venous abscess with the skin fistula showed gradual progression at 3 months postoperatively. The causal relationship between phlebitis and foreign body granuloma has not yet been clarified; however, a relatively long follow-up period may be required to detect the late development of foreign body granuloma, particularly for cases like ours with late phlebitis.

In the present case, clinical and ultrasound findings revealed no abnormal dilatation suggestive of a venous abscess in other crural and femoral lesions. To minimize the risk of postoperative saphenous neuropathy, localized removal of the abscess lesion was performed. To date, that is, 1.5 years postoperatively, no signs of recurrence of abscess formation have been observed in other areas. Periodical follow-up with ultrasonography is planned to monitor for potential recurrence.

## Conclusion

The postoperative course following CAE is uneventful in most cases, and postoperative complications such as those observed in our case are uncommon but may require surgical interventions. Predictive factors remain uncertain; therefore, careful follow-up is necessary to detect late complications following CAE, particularly in patients with underlying immunologic disorders.
